# Supplementary material for: Massively parallel assessment of gene regulatory activity at human cortical structure associated variants
Source: bioRxiv. 2025 Feb 13:2025.02.08.635393. Preprint. [Version 2] doi: 10.1101/2025.02.08.635393 (PMC11839127; doi:10.1101/2025.02.08.635393)
Supplement: Supplement 1 [file media-1.pdf]

Supplementary Information

Supplementary Figures..1
Supplementary Figure 1| Reproducibility of caQTL.. 1
Supplementary Figure 2| Examples of barcodes with outlier expression................................ 2
Supplementary Figure 3| Barcode representation in MPRA element..................................... 3
Supplementary Figure 4| Positive controls show higher MPRA activity than negative controls 4
Supplementary Figure 5| Limited concordance between exogenous multiplexed reporter and endogenous chromatin accessibility assays..5
Supplementary Figure 6| Overview of MPRA elements and cortical structure associated loci. 7
Supplementary Figure 7| Negative controls with a B-Box-like sequence show MPRA activity. 9
Supplementary Figure 8| Projection of cortical regions between atlases............................. 10
Supplementary Figure 9| TFs associated with MPRA activity in the stimulated condition..... 11
Supplementary Tables..12
Supplementary Table 1| Summary of the tested elements after QC....................................12
Supplementary Table 4| Summary of correlation between TF expression level across brain area and GWAS effect size (detailed statistics are provided in Supplementary Table 5)...... 13
Supplementary Table 5| Correlation between TF expression level across cortical areas and GWAS effect size (Related to Supplementary Table 4).. 13
Supplementary Table 6| Primer information for oligo library.. 14
Supplementary Table 7| Primer information for barcode mapping...14
Supplementary Table 8| Primer information for MPRA sequencing.......................................14
Supplementary Table 9| CRISPRi screening gRNA sequences.. 15
Supplementary Table 10| qPCR primers for CRISPRi screening.. 15
References.. 15

Supplementary Figures

Supplementary Figure 1| Reproducibility of caQTL

We compared the effect sizes of caQTLs identified in a population or primary human neural progenitors from two separate differentiations of largely the same donor lines derived from standard media[1] or media including a vehicle control[2]. Open circles indicate all caQTLs identified by previous study[2], and circles in red indicate caQTLs used in this MPRA study which were selected such that the caSNP was within the caPeak. The effect sizes are strongly correlated between studies ($r = 0.87$, $p < 2.2 \times 10^{-16}$ for all caQTLs and $r = 0.98 < 2.2 \times 10^{-16}$ for the caQTLs used in the MPRA, respectively), indicating that the caQTLs were highly reproducible.

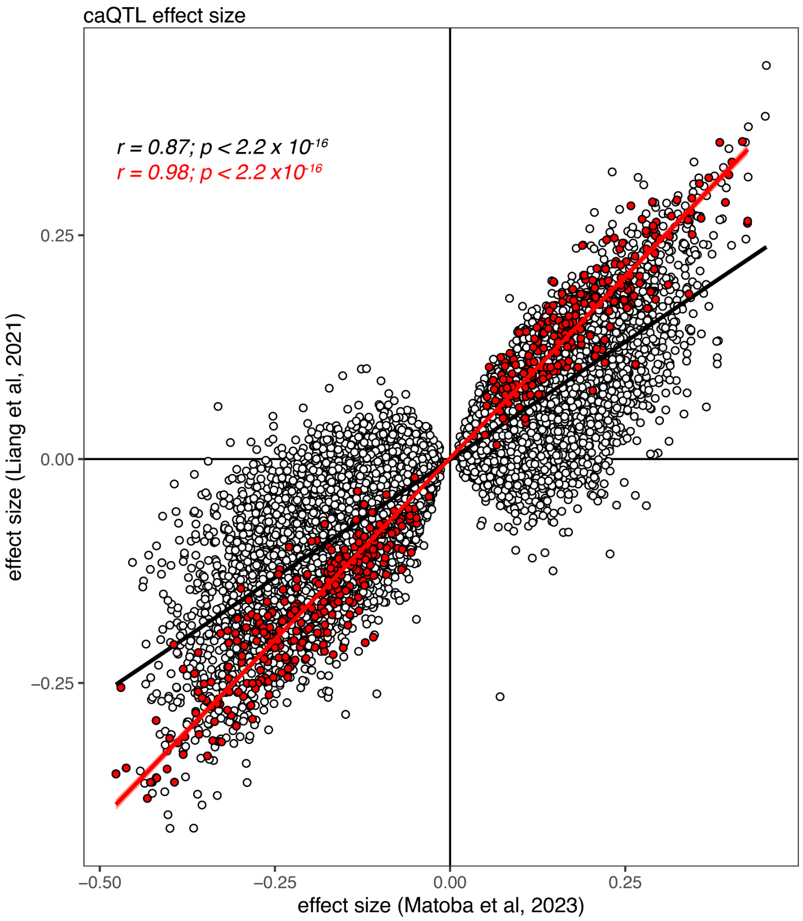

Supplementary Figure 2| Examples of barcodes with outlier expression

Barcode outliers were identified by *boxplot.stats(log2(RNA/DNA))* function with the default setting in each variant within a replicate (**a**). We removed those outliers across replicates (**b**). Triangles in red represent outliers found in the replicate, and black represent outliers found in other replicate(s). Three examples from (1) positive control, (2) negative control, and (3) ENIGMA datasets are shown.

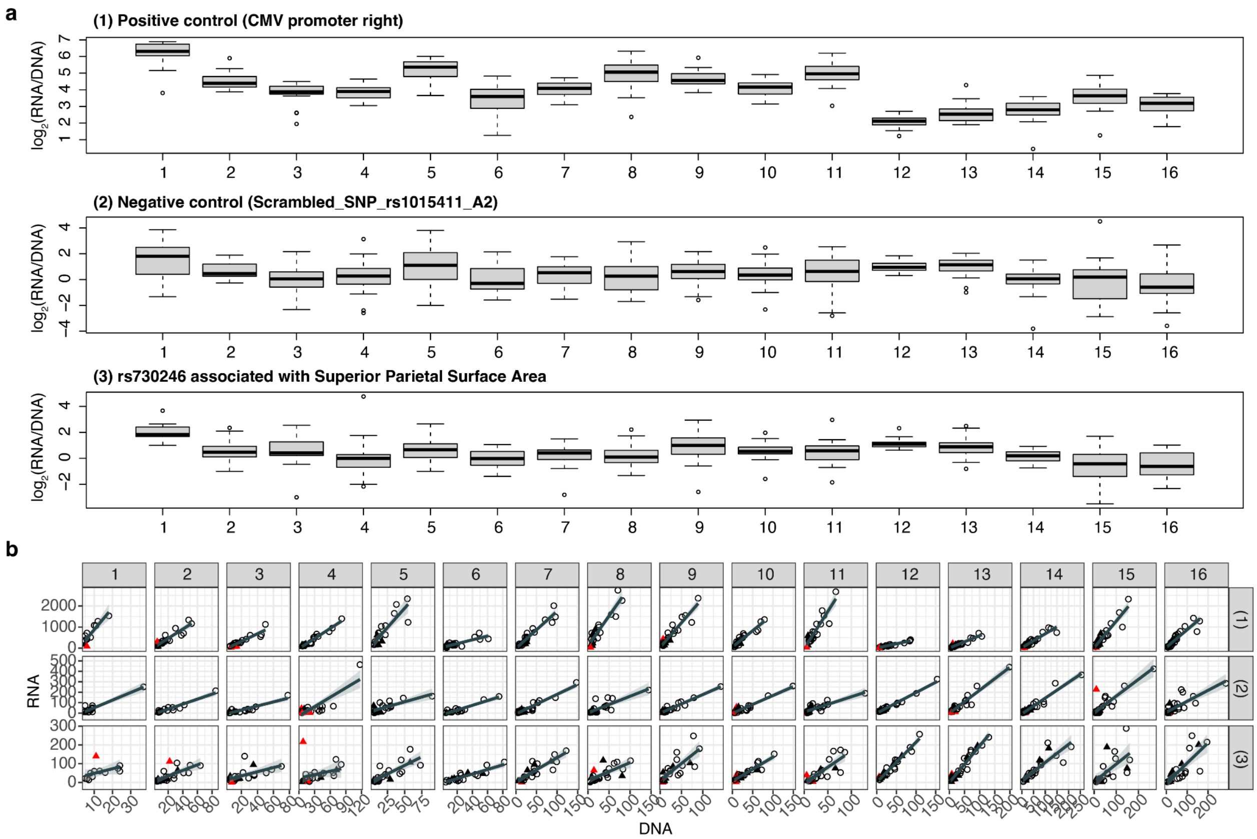

Supplementary Figure 3| Barcode representation in MPRA element

For elements which survived quality control filtering (barcode representation > 5, present in replicates > 2, removal of barcode outliers based on activity), histograms show barcode count per element in vehicle condition (across element median = 28, (s.d.) = 12.2) (**a**) and stimulated condition (across element median = 29 (s.d. = 12.7) (**b**).

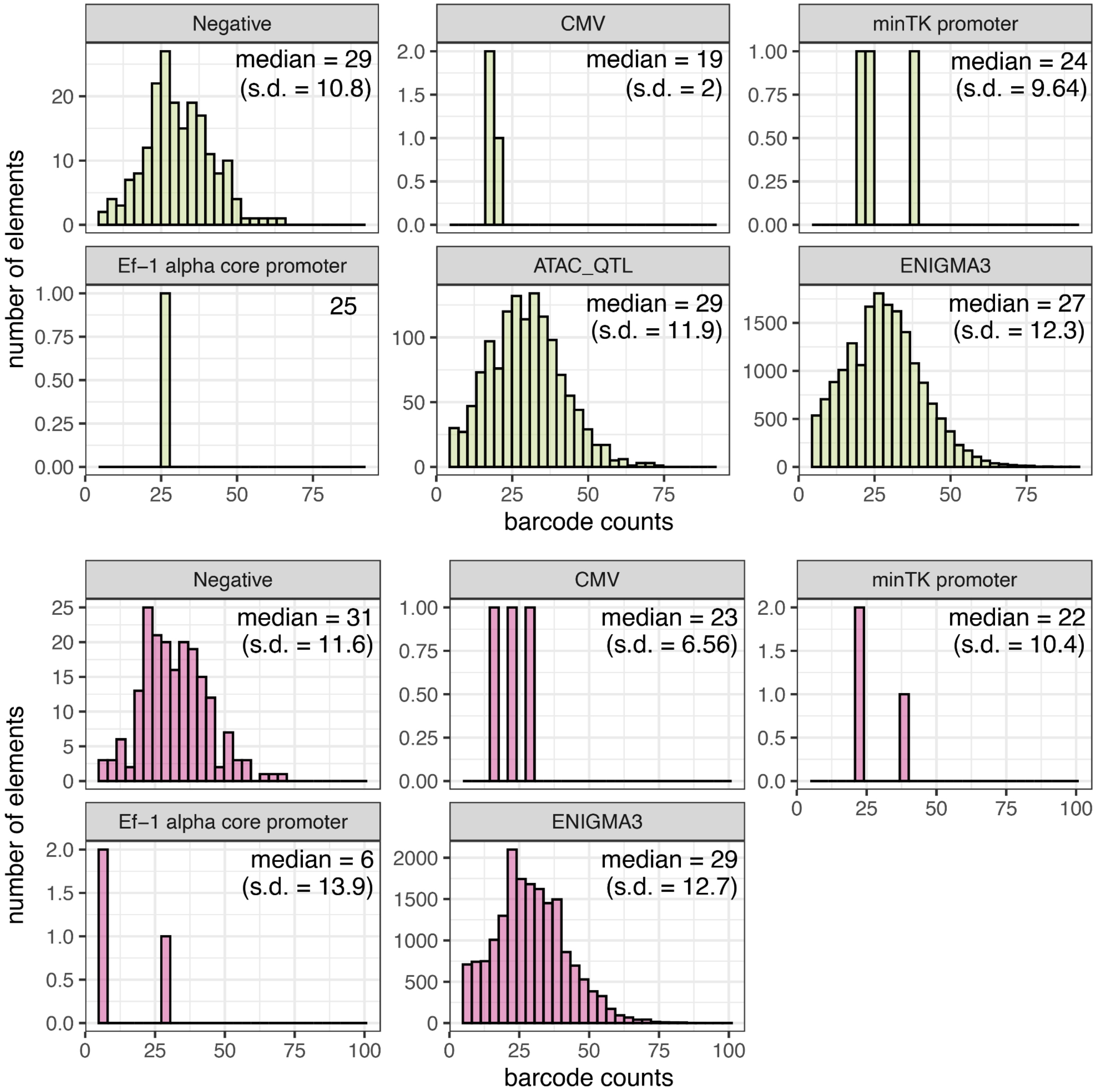

Supplementary Figure 4| Positive controls show higher MPRA activity than negative controls

Scatter plot of DNA vs RNA counts of positive controls (colored in red) and negative controls (colored in blue) in replicate 1.

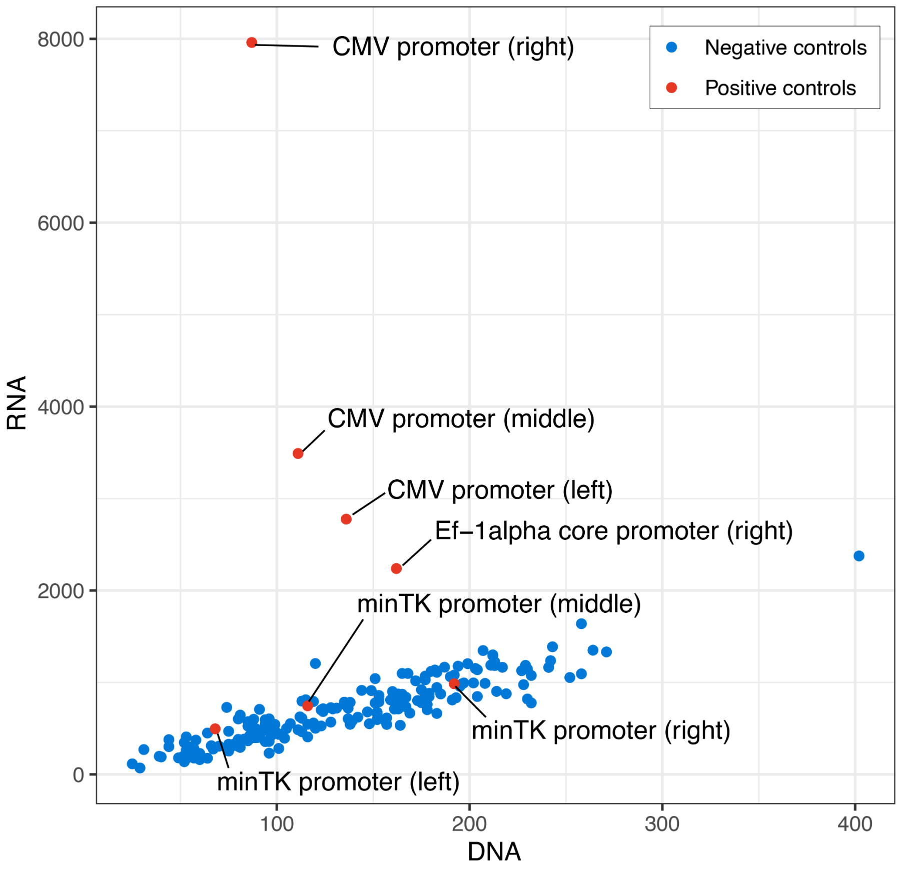

Supplementary Figure 5| Limited concordance between exogenous multiplexed reporter and endogenous chromatin accessibility assays

a. Features correlated with caQTL MPRA active elements. Nominal p-values calculated by two-sided Wilcoxon Rank Sum test are given in the boxes, and the color corresponds to the signed $-\log_{10}$(p-value). The number of SNPs in high LD ($r^2 > 0.6$) with the caSNP within the 150 bp insert was significantly associated with MPRA activity, suggesting that haplotype effects might combinatorially increase MPRA activity, as has been found in previous work[3]. We also detected a nominally significant positive relationship between GC content of the insert and depth of ATAC coverage with the level of MPRA activity, suggesting that sequence biases or the proportion of cells with accessible regions lead to increased MPRA activity. The percentage of correlated peaks was also associated with decreased MPRA activity, suggesting that some regulatory elements cannot influence transcription on their own and instead must work together in networks to influence transcriptional activity. **b.** MPRA allelic effects ranked by effect size of the chromatin accessibility increasing allele compared with caQTL effect sizes. 6% of caQTL elements showed allelic effects in the same direction as MPRA (emVar(+), right) and 6% of caQTL elements showed allelic effects in the opposite direction as MPRA (emVar(-), left). **c.** No significant difference in caQTL effect size was detected between regions with MPRA allelic effects and regions without significant allelic effects. **d.** We detected no relationship between the allelic effect size in MPRA and the allelic effect size in caQTL. Colors indicate emVar(+) (red) or emVar(-) (blue) elements. **e-f**. Representative caQTLs for emVar(-) **(e)** and MPRA*+ **(f)** elements are shown. MPRA element (one per Locus) : $n_{\text{MPRA active}} = 30$, $n_{\text{MPRA inactive}} = 472$, $n_{\text{emVar++}} = 30$, $n_{\text{emVar-*-}} = 30$, $n_{\text{MPRAn.s\_allelic}} = 439$. $n_{\text{replicate}} = 14$.

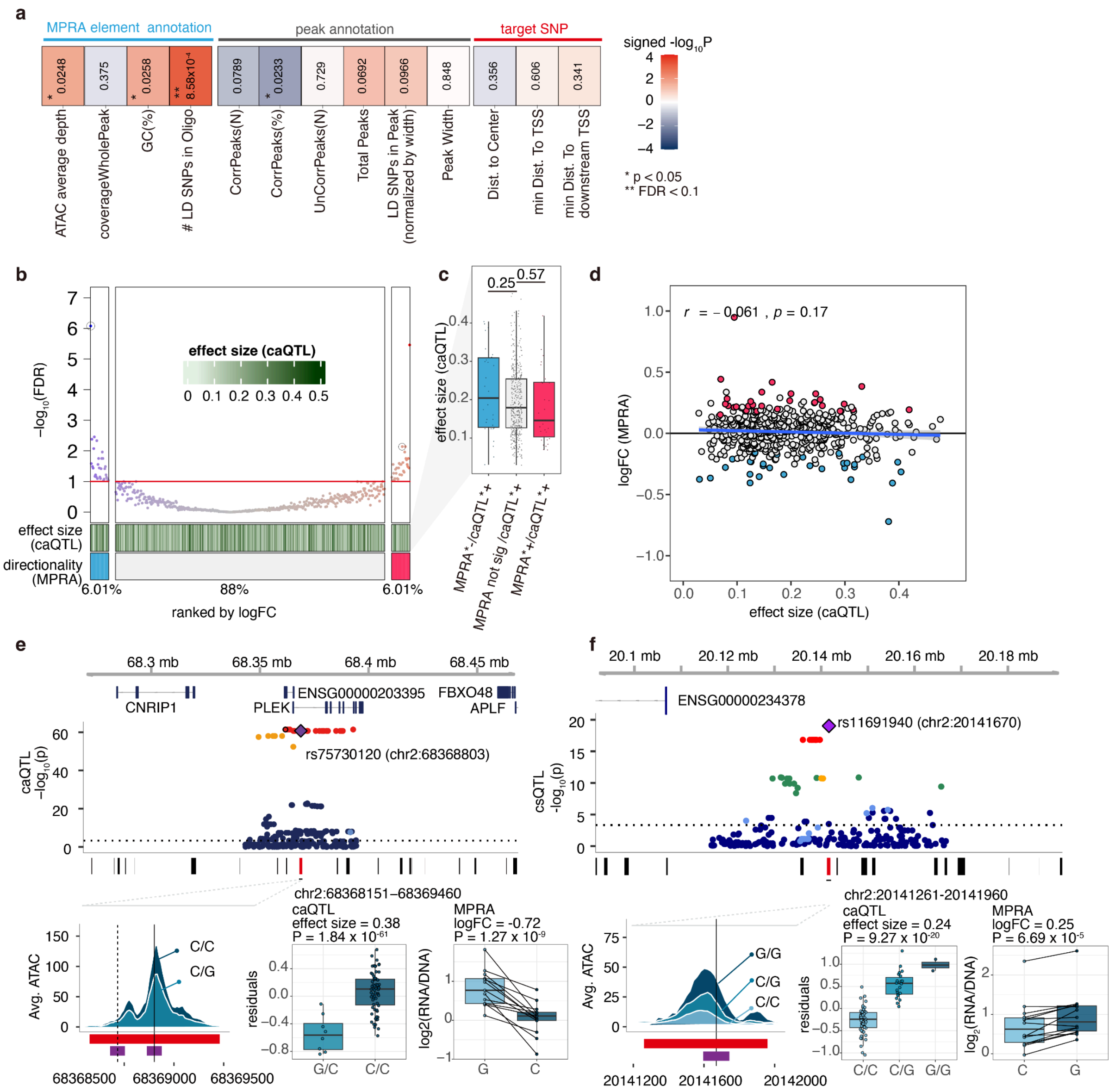

a
MPRA element annotation
peak annotation
target SNP
signed -log10P
* p < 0.05
** FDR < 0.1
ATAC average depth
coverageWholePeak
GC(%)
# LD SNPs in Oligo
CorrPeaks(N)
CorrPeaks(%)
UnCorrPeaks(N)
Total Peaks
LD SNPs in Peak (normalized by width)
Peak Width
Dist. to Center
min Dist. To TSS
min Dist. To downstream TSS
b
effect size (caQTL)
−log10(FDR)
directionality (MPRA)
6.01%
88%
6.01%
ranked by logFC
c
effect size (caQTL)
MPRA*-/caQTL*+
MPRA not sig /caQTL*+
MPRA*+/caQTL*+
d
r = −0.061 , p = 0.17
logFC (MPRA)
effect size (caQTL)
e
CNRIP1
ENSG00000203395
FBXO48
PLEK
APLF
rs75730120 (chr2:68368803)
caQTL −log10(p)
chr2:68368151−68369460
caQTL
effect size = 0.38
P = 1.84 x 10-61
MPRA
logFC = -0.72
P = 1.27 x 10-9
Avg. ATAC
residuals
log2(RNA/DNA)
f
ENSG00000234378
rs11691940 (chr2:20141670)
csQTL −log10(p)
chr2:20141261-20141960
caQTL
effect size = 0.24
P = 9.27 x 10-20
MPRA
logFC = 0.25
P = 6.69 x 10-5

Supplementary Figure 6| Overview of MPRA elements and cortical structure associated loci

a. UpsetR plot displays shared elements assessed for MPRA activity (top) and identified as MPRA active elements (bottom). Numbers in the barplot indicate element counts for either vehicle or stimulated condition (union) and each condition (vehicle, stimulation, respectively). Next to the UpsetR plot, the percentages of genomic features of 150 bp around the variant and allelic availability are shown. Genomic features are prioritized as follows: Exons > intron-exon boundaries > promoters > 1 to 5 kb from transcription start site [TSS] > introns > intergenic. **b**. Cortical-structure-associated loci ranked by the number of MPRA elements tested within the locus (top). Colors in heatmaps indicate phenotype-specific or shared locus where at least one MPRA variant is active stratified by condition. **c**. Histograms show the number of phenotype-specific loci (N = 55) and shared loci containing at least one MPRA active element. TH-SA: Thickness and surface area phenotypes; TH: Thickness; SA: Surface area.

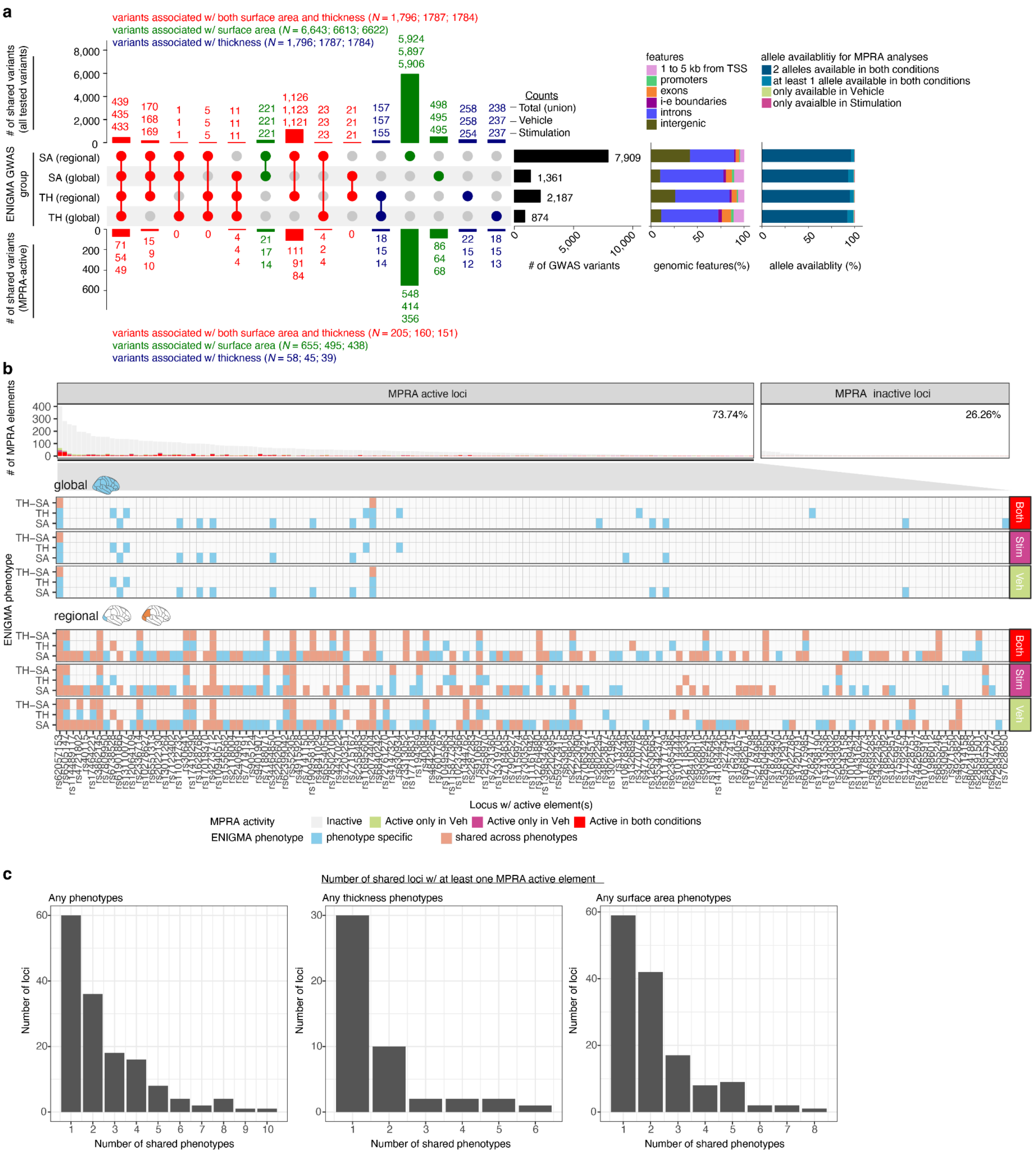
a
variants associated w/ both surface area and thickness (N = 1,796; 1787; 1784)
variants associated w/ surface area (N = 6,643; 6613; 6622)
variants associated w/ thickness (N = 1,796; 1787; 1784)
# of shared variants (all tested variants)
Counts
Total (union)
Vehicle
Stimulation
ENIGMA GWAS group
SA (regional)
SA (global)
TH (regional)
TH (global)
7,909
1,361
2,187
874
# of GWAS variants
features
1 to 5 kb from TSS
promoters
exons
i-e boundaries
introns
intergenic
genomic features(%)
allele availablitiy for MPRA analyses
2 alleles available in both conditions
at least 1 allele available in both conditions
only available in Vehicle
only avaialble in Stimulation
allele availablity (%)
# of shared variants (MPRA-active)
variants associated w/ both surface area and thickness (N = 205; 160; 151)
variants associated w/ surface area (N = 655; 495; 438)
variants associated w/ thickness (N = 58; 45; 39)
b
MPRA active loci
73.74%
MPRA inactive loci
26.26%
# of MPRA elements
global
regional
ENIGMA phenotype
TH–SA
TH
SA
Both
Stim
Veh
Locus w/ active element(s)
MPRA activity
Inactive
Active only in Veh
Active only in Veh
Active in both conditions
ENIGMA phenotype
phenotype specific
shared across phenotypes
c
Number of shared loci w/ at least one MPRA active element
Any phenotypes
Any thickness phenotypes
Any surface area phenotypes
Number of loci
Number of shared phenotypes

Supplementary Figure 7| Negative controls with a B-Box-like sequence show MPRA activity

Histograms of MPRA activity of negative controls quantified as transcription rate. Scrambled sequences generated from the rs73004638 region contain a B-Box-like sequence (GTTCGAGAC) (A1 = A, A2 = T at position 76), and show high transcription rate.

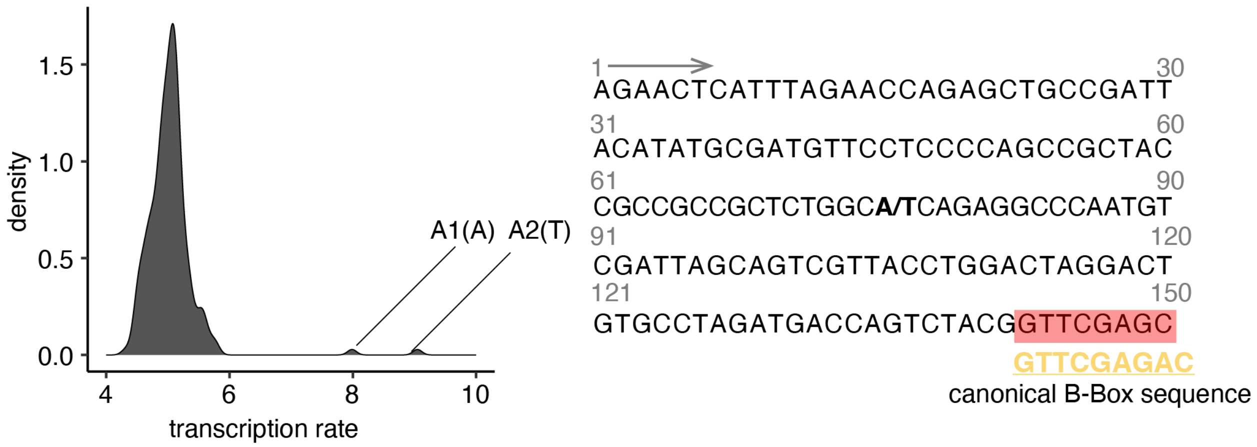

Supplementary Figure 8| Projection of cortical regions between atlases

34 cortical regions from Desikan-Killiany Atlas (DK atlas) used in the ENIGMA dataset were mapped to 16 macroscale regions defined by The Allen Human Brain Atlas (AHBA).

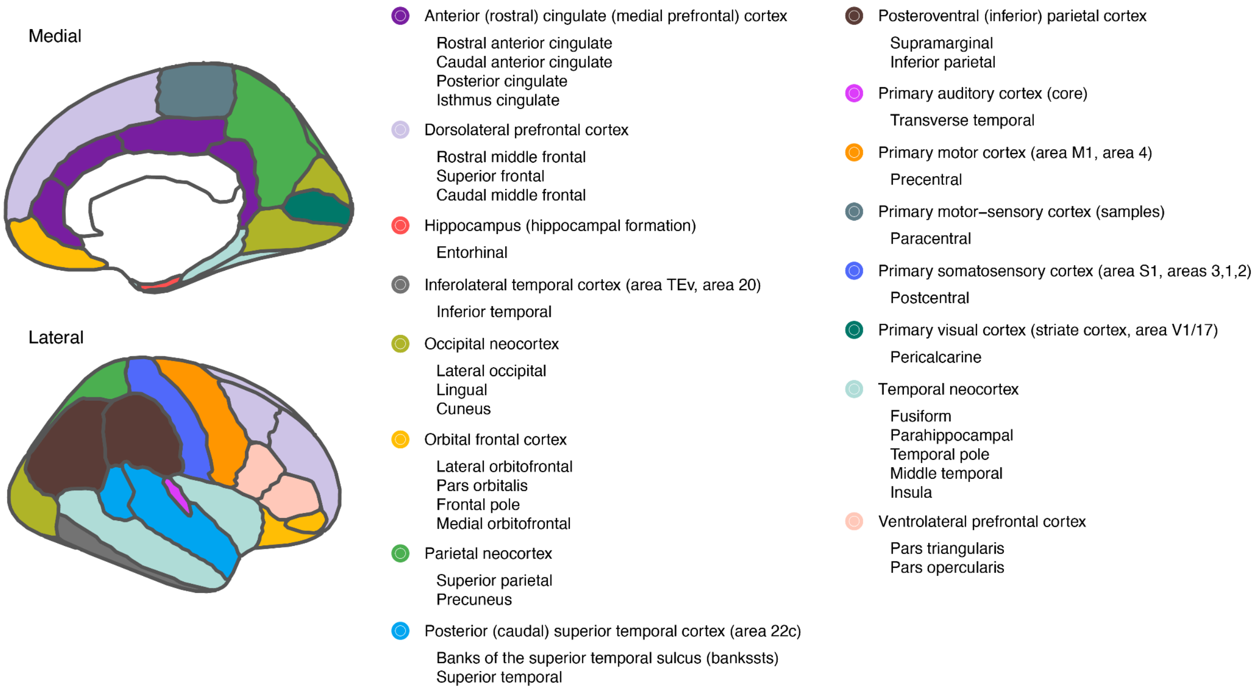

Supplementary Figure 9| TFs associated with MPRA activity in the stimulated condition

a, Proportion of elements containing TF binding motifs stratified by MPRA activity. **b**, Enriched TF binding clusters. (**a**,**b**) Statistical significance of enrichment was assessed by a two-sided Fisher's exact test. **c**, The level of TF motif disruption (x-axis) is related to MPRA allelic differences (y-axis). P-values were estimated by Pearson's correlation test.

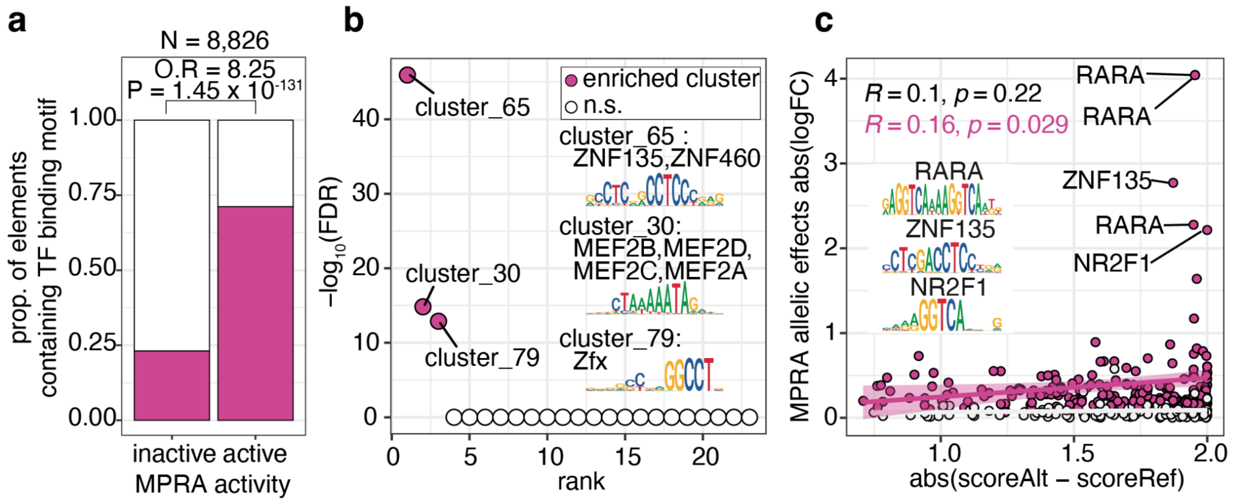

Supplementary Tables

Supplementary Tables 2 and 3 are provided in Excel files.

Supplementary Table 1| Summary of the tested elements after QC

		caQTL		ENIGMA					
		all	paired	all			paired		
	Condition	Veh	Veh	Veh	Stim	Union	Veh	Stim	Union
A	Elements	1,316	1,310	17,837	17,848	17,980	17,570	17,592	17,756
B	Variants	661	655	9,052	9,052	9,092	8,785	8,796	8,878
C	Locus	502	499	198	198	198	198	198	198
D	Active elements	37	34	988	927	1,258	949	897	1,202
E	Active variants	33	30	700	628	918	661	598	863
F	Active locus	30	28	138	133	150	135	131	146
G	% active elements (D/A)	2.81	2.60	5.54	5.19	7.00	5.40	5.10	6.77
H	% active variants (E/B)	4.99	4.58	7.73	6.94	10.10	7.52	6.80	9.72
I	% active Locus (F/C)	5.98	5.61	69.70	67.17	75.76	68.18	66.16	73.74
J	emVars		13				386	333	536
K	Locus with emVar(s)		12				107	107	126
L	% emVars (J/E)		43.33				58.40	55.69	62.11
M	% locus with emVar(s) (K/F)		42.8				79.26	81.68	86.30

Supplementary Table 4| Summary of correlation between TF expression level across brain area and GWAS effect size (detailed statistics are provided in Supplementary Table 5)

GWAS measurement	SNP disrupts motifs	Number of TFs	TFs (coefficient; BH-adjusted P)
Surface area	rs12046466	2	RARG (1.34; 0.00611)<br>SREBF1 (1.34; 0.0465)
Surface area	rs1910326	4	EOMES (−1.59; 0.0688)<br>SNAI3 (1.61; 0.0637)<br>TBX15 (−1.41; 0.0061)<br>TWIST1 (1.97; 0.0061)
Surface area	rs2395882	1	ZNF85 (−0.845; 0.0686)
Surface area	rs28735393	3	CREB1 (−1.25; 0.0686)<br>RARG (−1.66; 0.000483)<br>RORB (1.34; 0.0686)
Thickness	rs11887401	1	FOSL2 (−1.16; 0.0275)
Thickness	rs28495472	1	SREBF2 (1.02; 0.0688)
Thickness	rs62064600	1	SREBF2 (1.29; 0.0768)

Supplementary Table 5| Correlation between TF expression level across cortical areas and GWAS effect size (Related to Supplementary Table 4)

GWAS measurement	SNP	TF	coef	SE	t value	p_value	BH-adjusted-P
Surface area	rs12046466	RARG	1.34	0.277	4.84	3.13×10^{-5}	0.0061
Surface area	rs12046466	SREBF1	1.34	0.339	3.96	3.97×10^{-4}	0.0465
Surface area	rs1910326	EOMES	-1.59	0.451	-3.53	1.29×10^{-5}	0.0688
Surface area	rs1910326	SNAI3	1.61	0.425	3.78	6.52×10^{-4}	0.0637
Surface area	rs1910326	TBX15	-1.41	0.422	-3.33	2.18×10^{-3}	0.0984
Surface area	rs1910326	TWIST1	1.97	0.395	4.98	2.14×10^{-5}	0.0061
Surface area	rs2395882	ZNF85	-0.85	0.233	-3.62	9.96×10^{-4}	0.0686
Surface area	rs28735393	CREB1	-1.25	0.345	-3.63	9.79×10^{-4}	0.0686
Surface area	rs28735393	RARG	-1.66	0.272	-6.09	8.25×10^{-7}	0.00048
Surface area	rs28735393	RORB	1.34	0.371	3.60	1.05×10^{-3}	0.0686
Thickness	rs11887401	FOSL2	-1.16	0.275	-4.22	1.88×10^{-4}	0.0275
Thickness	rs28495472	SREBF2	1.02	0.29	3.53	1.29×10^{-3}	0.0888
Thickness	rs62064600	SREBF2	1.29	0.374	3.46	1.57×10^{-3}	0.0768

Supplementary Table 6| Primer information for oligo library

Primer	Sequence
MPRA-chipprimer-F	TATGCTGGTATACGCCTACA
MPRA-chipprimer-R	CCGTCACTAACTAACAGTGG
MPRA-BC_Primer_R	/5BiosG/AGTCGACTAGTNNNNNNNNNNNNNNNNNNNNTCTAGACCGT CACTAACTAACAGTGG

Supplementary Table 7| Primer information for barcode mapping

Primer	Sequence
Bcmap_P5_AAV_R	AATGATACGGCGACCACCGAGATCTACACCGATAAGCTTGATATCGA ATTCCC
Bcmap_P7_AAV_F_index	CAAGCAGAAGACGGCATACGAGATNNNNNNATTATCACTAGGGGTT CCTGC
BCmap_R1Seq_AAV_R	CGATAAGCTTGATATCGAATTCCCTAATGCACTAGT
BCmap_R2Seq_AAV_F	ATTATCACTAGGGGTTCCTGCGGCCGCA
BC_Map_R1seq	GTAACCACCCTGATCGACGGGGAGTGTACTAGT

Supplementary Table 8| Primer information for MPRA sequencing

Primer	Sequence
Lib_Hand_RT	ATGCTCTTCCCAACTGCCGACGGGGAGTGTACTAGT
Lib_Hand	TGCTCTTCCCAACTGCCGA
Lib_seq_Luc_R	TACAACCGCCAAGAAGCTGC
P5_seq_Luc_F	AATGATACGGCGACCACCGAGATCTACACTACAACCGCCAAGAAGCTGC
P7_Ind_#_Han	CAAGCAGAAGACGGCATACGAGATNNNNNNNNGCGTGCTCTACGACTATGCTCTT CCCAACTGCCGA
Index_primer	TCGGCAGTTGGGAAGAGCATAGTCGTAGAGCACGC
R1_primer	CCAAGAAGGGCGGCAAGATCGCCGTGTAATAATTCTAGA

Supplementary Table 9| CRISPRi screening gRNA sequences

Name	Position	Strand	Sequence (5 ' - 3')
Control-1	NA	NA	GAACCTCCCCGAATATCTGG
Control-2	NA	NA	GTATTACTGATATTGGTGGG
FOXO3-RE2-76	chr6:108,927,356	+	GGAATTCTGTACAGTATGCTGGG
FOXO3-RE2-259	chr6:108,927,539	+	CCTTCCCAAGGGCGGTTTGTTGG
**FOXO3-RE2-263**	**chr6:108,927,543**	**-**	**CTTGCCAACAAACCGCCCTTGGG**
**FOXO3-RE2-474**	**chr6:108,927,754**	**+**	**TATGATTAAGGCTTATCTACAGG**

Supplementary Table 10| qPCR primers for CRISPRi screening

Target gene	Strand	Sequence (5 ' - 3')
ACTB	+	CATGTACGTTGCTATCCAGGC
ACTB	-	CTCCTTAATGTCACGCACGAT
FOXO3	+	CGGACAAACGGCTCACTCT
FOXO3	-	GGACCCGCATGAATCGACTAT

References

1. Liang, D. *et al.* Cell-type-specific effects of genetic variation on chromatin accessibility during human neuronal differentiation. *Nat. Neurosci.* (2021) doi:10.1038/s41593-021-00858-w.
2. Matoba, N. *et al.* Stimulating Wnt signaling reveals context-dependent genetic effects on gene regulation in primary human neural progenitors. *Nat. Neurosci.* **27**, 2430–2442 (2024).
3. Abell, N. S. *et al.* Multiple causal variants underlie genetic associations in humans. *Science* **375**, 1247–1254 (2022).
